# Mechanisms of Dysregulated Humoral and Cellular Immunity by SARS-CoV-2

**DOI:** 10.3390/pathogens9121027

**Published:** 2020-12-08

**Authors:** Nima Taefehshokr, Sina Taefehshokr, Bryan Heit

**Affiliations:** 1 Department of Microbiology and Immunology, Center for Human Immunology, The University of Western Ontario, London, ON N0M 2N0, Canada; ntaefehs@uwo.ca; 2 Immunology Research Center, Tabriz University of Medical Sciences, Tabriz 51368, Iran; sinataefehshokr@gmail.com; 3 Robarts Research Institute, London, ON N6A 5K8, Canada

**Keywords:** COVID-19, SARS-CoV-2, adaptive immunity, B and T cell immune response

## Abstract

The current coronavirus disease 2019 (COVID-19) pandemic, a disease caused by severe acute respiratory syndrome corona virus 2 (SARS-CoV-2), was first identified in December 2019 in China, and has led to thousands of mortalities globally each day. While the innate immune response serves as the first line of defense, viral clearance requires activation of adaptive immunity, which employs B and T cells to provide sanitizing immunity. SARS-CoV-2 has a potent arsenal of mechanisms used to counter this adaptive immune response through processes, such as T cells depletion and T cell exhaustion. These phenomena are most often observed in severe SARS-CoV-2 patients, pointing towards a link between T cell function and disease severity. Moreover, neutralizing antibody titers and memory B cell responses may be short lived in many SARS-CoV-2 patients, potentially exposing these patients to re-infection. In this review, we discuss our current understanding of B and T cells immune responses and activity in SARS-CoV-2 pathogenesis.

## 1. Introduction

The severe acute respiratory syndrome corona virus 2 (SARS-CoV-2) was first identified in late December 2019, in Wuhan, China, and was first recognized as viral pneumonia from an unspecified infectious agent [1]. On 7 January 2020, the Chinese center for disease control and prevention reported the virus as a novel coronavirus from an infected patient’s throat swab sample [2]. On 23 January 2020, the World Health Organization declared the disease a public health emergency of international concern, and on 12 February 2020 officially named the causative virus SARS-CoV-2 and the resulting disease as coronavirus disease 2019 (COVID-19) [3]. Coronaviruses have caused two previous major outbreaks—severe acute respiratory syndrome (SARS-CoV) in 2002–2004, and Middle East respiratory syndrome (MERS-CoV) in 2012 [4]. An additional four coronaviruses are the cause of endemic infections in humans (229E, NL63, OC43, and HKU1), accounting for 15–30% of common cold cases [5]. Unlike the endemic coronaviruses, COVID-19 infection has a high case fatality rate (CFR 2.2% globally) [6] and is characterized by three vital symptoms—fever over 38 °C, dyspnea, and dry cough [7].

Coronaviruses (CoVs) are enveloped, crownlike, positive-stranded RNA viruses (26–32 kilobases), which belong to the family of Coronaviridae [8]. The genome structure of SARS-CoV-2 is similar to SARS-CoV, and includes four structural proteins, membrane (M), envelope (E), spike (S), and nucleocapsid (N), and 14 open reading frames (ORFs) in which ORF1ab encode 16 nonstructural proteins (NSPs) [9]. SARS-CoV-2 spike (S) protein enables infection by binding angiotensin-converting enzyme 2 receptor (ACE2) on host cells. Following binding, the S-protein is cleaved and activated by host transmembrane protease, serine 2 (TMPRSS2), allowing for viral entry to occur [10]. A recent study showed that scavenger receptor B type I, which normally binds to high density lipoprotein [11], binds to the S1 subunit of SARS-2 S protein to aid in viral entry into the host cell. This receptor is co-expressed with ACE2 in human pulmonary tissue and enhances SARS-CoV-2 infectivity through an HDL-dependent mechanism [12]. Many of the other coronaviral proteins, including N, M, NSP1, NSP3b, NSP4a, NSP4b, and NSP15, are involved in the modulation of the host immune response and immune evasion [13]. These immunoevasory mechanisms include suppression of antigen presentation on major histocompatibility complex (MHC) class I and II, inhibition of interferon (IFN) production and signaling, and exhausting natural killer (NK) cell-mediated cytotoxicity. These mechanisms have recently been reviewed in detail by Taefehshokr et al. [14].

Interstitial pneumonia and lymphopenia, driven by high levels of pro-inflammatory cytokines including interleukin 6 (IL-6) and tumor necrosis factor alpha (TNF-α), C-X-C ligand 10 (CXCL10), C-C motif ligand 2 (CCL2) and CCL3, may be seen in severe cases. This cytokine storm plays a central role in the pathogenesis of COVID-19 and is responsible for significant morbidity in COVID-19 patients [15,16,17]. Adaptive immune responses in SARS-CoV-2 pathogenesis are only beginning to be understood and need to be understood to enable the development of vaccines and immunotherapy agents against SARS-CoV-2 [18,19]. Recently, the Food and Drug Administration issued an emergency use authorization for two monoclonal antibodies including casirivimab and imdevimab for treatment of mild COVID-19. These monoclonal antibodies are directed against S protein of SARS-CoV-2, blocking the virus attachment and entry into human cells [20]. The safety and effectiveness of these monoclonal therapies remains to be evaluated. Moreover, interim analysis of COVID-19 vaccines including Pfizer, Moderna and AstraZeneca showed 70–90% protective immunity without any severe side effects [21]. These COVID-19 vaccines target the trimeric S protein, which mediates host cell binding and entry and is the major target of neutralizing antibodies [22,23]. While these vaccines prevented symptomatic infection, their impact on asymptomatic infections has not yet been reported, nor it is known how long their protective immunity lasts, nor is their impact on SARS-CoV-2 transmission established. Future investigations will provide answers for these questions and will lead to effective vaccine strategies through involving both neutralizing antibodies and T cell immune responses.

Profound defects in T and B cell activation and function have been observed in COVID-19 patients [2,24]. COVID-19 patients show a substantial reduction in the numbers of peripheral CD4 and CD8 T cells, with the remaining cells exhibiting a hyperactivated status (CD38^+^ HLA-DR^+^) and are enriched for pro-inflammatory C-C chemokine receptor 6 (CCR6^+^) Th17 T cells. This suggests that T cell over-activation, mediated by high cytotoxicity of CD8^+^ cells and increased Th17 expression, partly accounts for severe immune injury in patients [25]. In severe SARS-CoV-2 patients, CD4^+^ T lymphocytes are rapidly activated and differentiated into pathogenic T helper (Th) 1 cells. These activated T cells together with inflammatory monocytes (CD14^+^CD16^+^ and high expression of IL-6) contribute to severe pulmonary syndrome in COVID-19 patients [26,27]. Thus, compromised innate immune function [14], and impaired adaptive immunity, frequently observed in elderly COVID-19 patients [28] could lead to more severe symptoms. In terms of sex differences and its correlation with the disease course of COVID-19, men exhibit higher pro-inflammatory plasma cytokines (IL-8 and IL-18) and non-classical monocytes (CD14^low^ CD16^+^) than women. Progressive disease in men was associated with higher age and poor CD8 T cell activation. Conversely, women experience robust HLA-DR-positive activated T cells in compared to men, and higher levels of innate immunity cytokines, such as IL-15 and TNF super family, member 10 correlate with disease progression in women [29]. This suggests that sex-biased differences could account for distinct mechanisms of COVID-19 disease progression, with men benefiting from increased T cell immune responses, whereas dampened innate immune activation might be more effective for women at early disease stages. This review will outline the critical role of adaptive immunity responses in SARS-CoV-2 infection based on the latest studies.

## 2. Overview of Adaptive Immune Responses

In general, T immune responses and neutralizing antibodies play a dominant role in the clearance of viral infections as the specific targeting of pathogen-derived antigens is required for the elimination of infected cells and neutralization of free virions, while the high specificity of the adaptive immune system enables a highly regulated and targeted immune response [30]. CD4^+^ T cells function predominantly by regulating the activity of CD8^+^ T cells and B cells, CD8^+^ T cells function predominantly via the targeted killing of virus-infected cells, while antibodies produced by B cells can block viral surface proteins and agglutinate virions, thereby preventing infection [31]. Effective adaptive immune responses against both B and T cell epitopes provide protective immunity, with these epitopes well established for the structural proteins of the related SARS-CoV and MERS-CoV [32].

## 3. B Cell Responses

B cells are critical for the clearance of most viral diseases, with most COVID-19 patients developing a humoral response within two weeks of infection [33,34]. Poor humoral responses are associated with ineffective SARS-CoV-2 clearance in some patients, further highlighting the importance of this response for viral clearance [35]. B cell development progresses linearly through pro-B cell, pre-B cell, and immature B cell states before giving rise to mature B cells. During B cell development, B cells rearrange their heavy and light chain loci through VDJ recombination. Several studies have demonstrated the biased usage of VDJ gene segments during the development of virus-specific antibodies. For instance, heavy chains encoded by IGHV3–30 (Immunoglobulin Heavy Variable 3–30) and IGHV3-21 are preferentially utilized during responses to development influenza and respiratory syncytial virus vaccines respectively [36,37]. IGHV3-21 gene pairing displayed neutralizing activity against respiratory syncytial virus despite lacking somatic mutations, this suggests that some pattern of recombination may induce protective antibody responses in young infants [36]. A similar pattern of biased VDJ recombination has been identified in some SARS-CoV-2 patients, with antibody development biased towards the immunoglobulin G (IgG) heavy chain IGHV3-23 and IGHV3-7—an observation which may be helpful for antibody production and design of SARS-CoV-2 vaccines [38]. It is unclear the extent to which these patterns of VDJ recombination vary between patients, and whether certain VDJ recombinants provide antibodies with superior SARS-CoV-2 neutralization capabilities or an increased propensity for pathological antibody-driven responses.

### 3.1. Pathological B Cell Polarization

Mature B cells that undergo class switching but lack expression of immunoglobulin D and the memory marker CD27 (IgD^−^ CD27^−^), are referred to as double-negative (DN) B cells [39]. Previous studies have shown that DN B cells are expanded in patients with rheumatoid arthritis, systemic lupus erythematosus (SLE), HIV, and other diseases with significant immune pathology. DN B cells, through cytokine release and autoantibody production, promote the progression of these diseases [40,41]. DN2 B cells (IgD^−^ CD27^−^ CXRC5^−^ CD21^+^), through an extra-follicular pathway that was previously identified in SLE [42], enhances COVID-19 pathogenesis at early disease stages [43]. In these cells activated toll like receptor 7 drives the expression of CD11c and T-bet, and through uncharacterized mechanisms, promotes severe COVID-19 disease [43]. Clearly, B cells responses can have negative effects on patient outcomes, but these mechanisms are poorly understood, and it is unclear the extent to which these deficits contribute to patients’ outcomes.

### 3.2. Antibody-Mediated Immune Response

Antibodies have a multifactorial role in viral clearance. Antibodies against viral entry receptors such as the SARS-CoV-2 S protein can sterically hinder interactions between the viral protein and it host-cell target, thereby preventing binding and subsequent infection [44]. Antibodies can also agglutinate freely circulating virions, allowing for their Fc-receptor mediated clearance [45]. Antibodies can also aid in the clearance of infected cells, with antibody binding to viral proteins expressed on the surface of the infected cell (e.g., during virion budding) directing NK cells to kill the infected cell via antibody-dependent cell cytotoxicity [46]. Lastly, the formation of memory B cells is a critical component of developing long-lasting immunity following viral clearance. But while antibodies are crucial for the clearance of viral pathogens, inappropriate activation of B cells may contribute to SARS-CoV-2 pathophysiology, and some antibodies may enhance infection [47].

Antibodies commonly bind the N and S proteins of SARS-CoV-2 [48,49,50]. The receptor binding domain (RBD) of the S protein has been shown to be highly immunogenic, and antibodies binding the RBD domain could potently block virus interactions with ACE2, thus neutralizing the virus [49,51]. Recent studies have identified potent neutralizing RBD-specific monoclonal antibodies in SARS-CoV-2 infection [49,52,53]. Given the high homology between the S protein of SARS-CoV and SARS-CoV-2 [54], there is a possibility of cross-reactivity of antibodies recognizing the S protein from SARS-CoV with SARS-CoV-2. However, using a pseudovirus system, convalescent sera from SARS-CoV patients were found to have limited cross-neutralization to SARS-CoV-2 pseudovirions, suggesting that recovery from SARS-CoV infection might not protect against SARS-CoV-2 infection, and that vaccine candidates for SARS-CoV are unlikely to be suitable for SARS-CoV-2 [54]. Additionally, anti SARS-CoV-2 antibodies or infected plasma did not show cross-reaction with SARS-CoV or MERS-CoV RBDs [49]. There is a correlation between antibody titers and disease severity, with patients whom experienced more severe disease having higher antibody titers than those who experienced asymptomatic or mild infection [55,56] However, this correlation appears to arise after disease resolution, with antibody titers during active disease showing no correlation to disease severity [57]. This suggests that severe disease may promote the formation of memory B cells, thus leading to higher post-infection antibody titers. Whether these higher titer levels provide additional protection to re-infection is currently unknown.

While neutralizing antibody responses are common against coronaviruses, the durability of these responses is often poor. For example, in response to SARS-CoV—the causative agent of the 2002 SARS outbreak—immunoglobulin M (IgM) antibody levels were observed to increase early in infection, with detectable increases occurring as early as 4 days after the onset of disease. IgG seroconversion occurred in most patients within 14 days of the onset of symptoms, but unlike with many other viruses, these neutralizing antibodies titers decreased dramatically 2–3 years after infection. While additional outbreaks of SARS have not occurred, this loss of neutralizing antibody titers implies that these patients would become susceptible to re-infection only a few years after the primary infection [58,59,60]. Indeed, this rapid decay of humoral immunity is common with coronaviruses, and the resulting re-infection of hosts is a major factor in the persistence of the endemic human coronaviruses [60,61]. In two preliminary study of SARS-CoV-2 patients, peak SARS-CoV-2-specific IgM was observed on day 9, with class-switching to IgG occurring by week 2 [62], with SARS-CoV-2 IgG titers beginning to decrease by 8 weeks post symptom onset [63]. This rate of antibody formation, isotype switching, and the subsequent decrease in antibody titres are typical of B cell responses to viral pathogens. In most cases the decrease in antibody titers ceases prior to pathogen-specific serum antibody concentrations dropping below protective levels. As such, it is unclear at this time whether this decrease in SARS-CoV-2 antibodies represent the typical course for antibody levels following pathogen clearance, or if it is indicative of the decrease to the non-protective levels often observed in SARS-CoV and MERS-CoV patients [64]. Preliminary studies have suggested that SARS-CoV-2-specific antibodies in some patients may only be maintained at protective levels for 2 months, indicating that humoral immunity to SARS-CoV-2 may be short-lived [65]. Similarly, a rapid decline of anti-SARS-CoV-2 antibodies has been observed in asymptomatic and mild COVID-19 patients, indicating that these individuals may have minimal protection against subsequent infections with SARS-CoV-2 [66,67]. Overall, despite the lower titers of neutralizing antibody at later timepoints in some individuals, neutralizing antibody titers may still be sufficient to provide protection from COVID-19 for some period. Although the required neutralizing antibody titer for protection from re-infection has not yet been established in humans, studies have shown that isolated neutralizing monoclonal antibodies from COVID-19 patients provided dose-dependent protection from infection in animal challenge models [52,68,69]. A recent study showed that two rhesus macaques that had resolved primary SARS-CoV-2 infection were resistant to re-infection 28 days later during recovering stage [70]. Similarly, in SARS-CoV-2 infected rhesus macaque model ~100 titers of neutralizing antibody were observed on day 35 when re-exposed against SARS-CoV-2 after the first infection, and no clinical signs of illness were seen following re-infection [71]. However, low level of virus was still detected in nasal swab after SARS-CoV-2 re-challenge, suggesting that the macaques had achieved immunologic control rather than sterilizing immunity against SARS-CoV-2. Whether these findings will translate to humans remains unclear, but the first trial of convalescent sera containing high titers of anti-SARS-CoV-2 antibodies showed no benefit to patients in a randomized control trial, suggesting that antibody-mediated immunity may not be sufficient for clearing SARS-CoV-2 [72].

### 3.3. Impaired and Pathological Antibody Responses in COVID-19

The durability and magnitude of antibody responses correlate with COVID-19 disease severity [48,49], and in cases of severe infection are often of low magnitude [73] and appear to lack durability [74]. This may be similar to SARS-CoV and MERS-CoV, where outside of a small subset of individuals, humoral responses typically dropped below protective titers within two years of infection [59,75]. A recent study showed that in severe COVID-19 patients, within 10 days of the onset of respiratory symptoms, splenic and lymph node germinal centers (GCs) and B cell lymphoma 6 (Bcl-6^+^) expressing B cells are markedly diminished. Lack of GCs accompanied by an absence of Bcl6 expressing B cells or Tfh cells suggests a mechanism for limited durability of antibody responses observed in SARS-CoV-2 infection [76]. While an antibody response was required to clear SARS-CoV-2 infection, patients who produced an early antibody response to the S protein had a higher risk of death and succumbed to the disease more quickly than those who developed antibody responses later in disease. This unexpected outcome was due to an increased polarization of alveolar macrophages to a pro-inflammatory M1 phenotype which increased inflammation and impaired healing within the lung [77,78]. This may represent antibody-dependent enhancement of disease (ADE), which has been reported for SARS-CoV and MERS-CoV, with early antibody production acting to enhancing viral entry into macrophages and other immune cells by enabling virion uptake via phagocytic Fc receptors [79,80,81]. The importance of the timing of this antibody response has profound implications in the use of convalescent sera or recombinant antibodies as a therapeutic approach, as the timing and dose of administration will likely affect patients’ outcomes. For example, early high titers of neutralizing antibodies are associated with more severe clinical outcomes in SARS-CoV-2 patients, indicating that antibody-based therapies may need to be used later in disease [82]. In conflict with this data is work from Eli Lilly which demonstrated that neutralizing antibodies only show efficacy if administered within 10 days of symptom onset [83]. Of greater concern is the implication of these findings for vaccine development, as vaccine efficacy may be limited by both the potential for developing short-lived memory, and from the risk of forming ADE-enhancing antibodies [84,85]. Indeed, ADE has plagued the development of other vaccines, including the Dengvaxia vaccine for dengue virus [86], and to-date, a satisfactory approach to preventing ADE has not been discovered.

Interestingly, a recent study revealed high neutralizing IgG auto-Abs against type I IFNs in nearly 15% of life-threatening COVID-19 patients [87], suggesting that the generation of auto-Abs in COVID-19 patients may be a risk factor for severe disease and mortality, and may account for the low serum IFNs levels observed in some patients with severe disease. In addition, a subset of severe COVID-19 patients have inborn errors of type I IFNs. These impairments in the IFN system likely contribute to T and B cell deficits in these patients, accounting for the increased pathophysiology they experience and delayed clearance of SARS-CoV-2 [88].

In addition to poor durability of immunity and the potential for ADE, recent evidence indicates that SARS-CoV-2 is likely to undergo antibody-driven evolution, allowing for escape from formerly neutralizing antibodies and higher upper respiratory viral load. This in vitro study suggested that D614G mutation could lead to increased transmissibility without measurable effect on infection outcome [89]. Supporting this possibility is the observation that in a large dataset of patient samples (25,000 whole-genome sequences from the UK) that a 614G mutation in the spike protein lead to increased viral loads in younger patients, and co-occurring mutations in neighboring sites (e.g., D614N) is suggestive that this region is under active evolutionary selection [90]. However, this observation is not universal, with a recent study analyzed 46,723 SARS-CoV-2 genome assemblies from 99 countries which found no evidence linking recurrent mutations with increased SARS-CoV-2 transmissibility, and also identified most mutations to be selectively neutral [91]. Due to the slow rate of SARS-CoV-2 evolution this escape is unlikely to occur within individual patients, but these mutations could lead to re-infection of individuals—even those who retain neutralizing antibody titers. Understanding these evolutionary processes, in particular predicting which mutations are selected in the presence of neutralizing antibodies, will be critical for the rational design of therapeutics and vaccines [92,93]. Moreover, a recent study mapped SARS-CoV-2 RBD mutations, it was indicated that mutations in receptor binding motif of the RBD—including at ACE2 contact residues—often lead to neutralizing antibody escape [94]. However, it remains unclear which mutations significantly affect the antigenicity of SARS-CoV-2.

## 4. T Cell Responses

CD8^+^ and CD4^+^ T cell immune responses from convalescent samples were extensively investigated in SARS-CoV and MERS-CoV patients, and early observations suggest a similar response occurs in SARS-CoV-2 patients. Similar to SARS-CoV, CD4^+^ and CD8^+^ T cells are reduced in the peripheral blood of SARS-CoV-2 patients, with the remaining T cells in highly activated state characterized by a large number of CD38^+^ HLA-DR^+^ cells [25,95]. The first report of the immune dynamics during mild COVID-19 demonstrated that in a single patient the number of circulating CD8^+^ T-cells (CD38^+^ HLA-DR^+^) and antibody secreting B cells increased early in disease, peaking 8 to 9 days after symptom onset. This peak was followed by a slow increase circulating T follicular helper (Tfh) cells up to the time of discharge, 20 days after symptom onset [96]. SARS-CoV S glycoprotein is the primary source of T cell epitopes, with lesser contributions from the M, N and ORF3 proteins [97]. In MERS-CoV-specific T cells, S, N and a small pool of E and M peptides accounted for T cell reactivity [98]. A recent study demonstrated that T cells could specifically recognize SARS-CoV-2 derived S proteins and at lower titers, a pool of other peptides including peptides from NSP3, NSP4, ORF3a and ORF8 [99,100]. Interestingly, a recent study identified T cell responses to only the S protein in seronegative subjects, while SARS-CoV-2 T cell response has been mounted to all other structural and accessory proteins in convalescent subjects [101]. This suggests that cross-reactive memory T cell responses against seasonal CoVs can be distinguished from patients who contracted COVID-19 due to the broad response against SARS-CoV-2 proteins versus a more limited response against seasonal CoV’s.

Unlike B cell memory, T cell memory against coronaviruses appears to be durable. SARS-CoV patients had reactivatable CD4^+^ and CD8^+^ T cells 6 years after infection [102], and this reactivity was specific as SARS-CoV reactivity could be transferred to other T cells through recombinant expression of the cloned TCR [103]. Immunization of animals with a MHC I restricted (e.g., CD8- T cell specific) peptide from the SARS-CoV-2 S protein led to a robust T cell response, indicating that the induction of viral-specific T cell responses by a vaccine is possible [104]. In animals, the adoptive transfer of SARS-CoV specific effector CD4 and CD8 T cells into severe combined immunodeficiency mice reduced virus titer in the lung and enhanced survival, even in the absence of B cells [104]. These data indicate that T cell responses are protective against coronaviral infection, and likely are important in the clearance of SARS-CoV-2. But while vaccination or adoptive transfer of SARS-CoV-specific CD4 and CD8 T cells could lead to the amelioration of clinical disease, it is still not clear whether infection-generated virus-specific T cells have an equally protective role against SARS-CoV-2.

While T cells appear to be protective against SARS-CoV-2, the virus may have mechanisms to limit T cell activation. Indeed, SARS-CoV-2 has been demonstrated to limit antigen presentation on both MHC I and MHC II, although the specific molecular mechanisms remain unclear [105,106,107]. Furthermore, several studies have shown that impaired respiratory DC (rDC) migration may act to limit antigen presentation and T cell priming in SARS-CoV patients [108,109,110]. CCR7 is required to direct the migration of rDCs to the draining lymph nodes, with this process inhibited by the presence of prostaglandin D2 (PGD2) [111,112]. It has been shown that PGD2 production is increased in SARS-CoV-infected mice, with this increase greater in aged animals, with this contributing to impaired rDCs migration and diminished virus-specific T cell responses in the lungs of SARS-CoV- infected mice [109]. It is unclear at this time whether SARS-CoV-2 has a similar effect on PGD2 production or on rDC migration.

### 4.1. Lymphopenia in COVID-19

Currently, much less is known about T cell fate in SARS-CoV-2 patients, and unlike MERS-CoV, it is unclear the extent to which SARS-CoV-2 can infect T cells and what the consequence of this infection is on T cell survival and activity [113]. Similar to severe SARS-CoV, progressive lymphopenia has been reported in SARS-CoV-2 patients [114,115]. Depletion is seen of both CD4^+^ and CD8^+^ frequency cells. This includes depletion of γδ-T cells [116] which provide protection against other viral infections including influenza [106,117,118]. Lymphopenia is thought to occur via splenic white pulp atrophy and decreased generation of lymphoid follicles, both of which have been observed in COVID-19 patient autopsies [119,120]. Circulating T cell frequencies return to normal in convalescent patients, with this recovery correlating to lower levels of circulating pro-inflammatory cytokines [121,122,123,124]. This indicates that lymphopenia may be driven, in part, by the cytokine storm which occurs during COVID-19, and consistent with this hypothesis, high circulating levels of IL-6 and IL-8 correlate with lymphopenia in COVID-19 patients [125].

It is unclear whether this lymphopenia is due to T cell death or retention outside of the circulation. For example, cytokines such as class I IFN’s and TNF-α could inhibit T cell recirculation in the blood through promoting retention in lymphoid organs and attachment to vascular endothelium [126,127]. At present, the link between cytokine release and lymphopenia remains correlative and it is unclear if this is a major contributor to lymphopenia. Cell death may also account for this lymphopenia, and indeed, in other viral diseases T cell lymphopenia is driven by IFN-induced apoptosis of memory CD8^+^CD44^high^ T cells. This apoptosis is induced by IFN-dependent expression of caspases 3 and 8, thus sensitizing the cells to apoptosis [128]. Necroptosis may also play a role in T cell death during viral infections, with studies demonstrating receptor-interacting serine/threonine-protein kinase 1 and 3 dependent necroptosis of T cells, although this only has been observed when caspase-8 and Fas-associated death domain protein is deleted from T cells [129,130,131]. The recently published interactome of SARS-CoV-2 showed that NSP12 interacts with receptor-interacting serine/threonine-protein kinase 1 (RIPK1), potentially allowing SARS-CoV-2 to regulate T cell necroptosis [132].

### 4.2. T Cell and NK Cell Exhaustion in COVID-19

While studies are suggestive of a successful CD8^+^/B/Tfh cell mediated clearance of mild SARS-CoV-2 infection, other investigations have shown that surviving T cells in SARS-CoV-2 patients are functionally exhausted and express high level of programmed cell death protein 1 (PD-1) and T-cell immunoglobulin mucin-3 (TIM-3) [123,133]. Both CD8^+^ and CD4^+^ T cells show other evidence of exhaustion in SARS-CoV-2 patients, in the form of decreased IFN-γ and IL-21 production [134,135]. This exhaustion may be transient, as PD-1 expression is reduced in recovered intensive care unit (ICU) patients compared to severe ICU patients [135].

Natural killer (NK) cells are essential in the control of viral infections, and the functional exhaustion of CD8^+^ T and NK cells is associated with the persistence of SARS-CoV-2 infection [134,136]. The expression of NK group 2, member A (NKG2A), an inhibitory receptor, on CD8^+^ T and NK cells leads to functional exhaustion of CD8^+^ T and NK cells in cancer and chronic viral infection [137,138]. Both NK cells and cytotoxic lymphocytes have increased expression of NKG2A and decreased expression of IFN-γ^+^, CD107a^+^, granzyme B^+^ and IL-2 in SARS-CoV-2 patients, consistent with functional exhaustion of these cells [2,134].

### 4.3. T Cell Contribution to the COVID-19 Cytokine Storm

The levels of pro-inflammatory cytokines may dictate the severity of COVID-19 disease, as highly elevated levels of IL-2, IL-7, G-CSF, MIP-1A, MCP-1, IP-10 and TNF-α were found in patients with severe SARS-CoV-2 infection. These findings were consistent with the cytokine profile of SARS-CoV and MERS-CoV, indicating the central role of a cytokine storm in the pathophysiology of COVID-19 [139,140]. In SARS-CoV-2 infection, the large quantities of circulating cytokines include IL-6 and IL-1β. This induces Th17 cell differentiation, promoting further IL-17 and IL-6 production and a worsening of the cytokine storm. Moreover, highly expanded clonal CD8^+^ T cells in the lung microenvironment of SARS-CoV-2 patients has suggested robust adaptive immune response in COVID-19 infection [141]. CD4^+^ and CD8^+^ T cells in COVID-19 patients gain increased capability to produce IL-17 in vitro, leading to inflammation and neutrophil activation [142]. Additionally, a recent study showed that monocytes-released IL-6, direct CD4 T cells in severe SARS-CoV-2 patients, and that this interaction is attenuated by treatment with an IL-6 inhibitor [27,143]. Based on these studies, blocking both IL-6 and IL-17 is a potential approach for controlling SARS-CoV-2 infection. This combinatorial approach is likely required, as targeting a single cytokine appears to be insufficient for treatment of COVID-19 [144]. These cytokines could then drive further lung inflammation and promote virus persistence by protecting virus-infected cells from apoptosis through inducing Bcl-2 and Bcl-xL expression [145,146].

Consistent with a role for IL-17, IL-17RA−/− mice had a reduced influx of neutrophils to the airways in response to pulmonary influenza infection, resulting in lower immune-related lung injury and higher survival rates [147]. On the other hand, SARS-CoV-2 has been observed to stimulate Th2 cytokine production, including IL-4 and IL-10, which would be expected to suppress Th1/Th17-mediated inflammation [139]. Similar patterns of cytokine production are observed in other coronaviral disease. For example, in SARS-CoV patients, a strong T cell immune response, indicated by elevated CD4^+^ and CD8^+^ T cell numbers, was correlated with higher neutralizing antibody activity and increased Th2 cytokines including IL-4, IL-5 and IL-10 [97,148]. Several SARS-CoV vaccine formulations, when used in animal models, resulted in enhanced immunopathology associated with Th2-mediated eosinophil infiltration [149,150]. Clearly, further investigation into Th17 versus Th2 polarization is required in SARS-CoV-2 patients, and therapies blocking IL-17 or Th2 responses may be of interest for the clinical management of COVID-19 patients.

Tfh cells, identified by their expression of the transcription factor Bcl-6, are a subset of CD4^+^ T cells that play critical role in the formation of GCs and in assisting B cell production of antibodies [151]. Increased numbers of Tfh cells have been shown in viral infections and following vaccinations, where they help to promote and maintain the production of virus-specific antibodies [152,153]. Studies have shown that bacterial infections and a lack of T follicular regulatory cells results in strong cytotoxic Tfh responses, correlating with lower GC and antibody response through increased GC B cell apoptosis [154,155]. Whether Tfh cells serve a similar role of SARS-CoV-2 infection is unclear, as some studies have identified increased cytotoxic Tfh numbers in COVID-19 patients, while others have identified decreased Bcl-6^+^ Tfh numbers and decreased GC counts in similar patients [133,156]. This absence of GCs correlated with increased Th1 responses and aberrant TNF-α production in lymph nodes [76]. In SARS-CoV infection, a similar loss of GCs and lymphoid depletion was also observed [157]. Impaired GC formation, through suppressed Tfh cell differentiation, has been observed in a mouse model of severe malaria, which could be reversed by TNF-α or IFN-γ blockade [158]. Similarly, *Ehrlichia muris* infection of mice caused a loss of GC in a TNF-α dependent manner [159]. Combined, these studies suggest that significant secretion of pro-inflammatory/Th1-type cytokines may negatively regulate Tfh cell differentiation, thus impairing the GC reactions that are required for the development of a strong and durable humoral response against SARS-CoV-2.

### 4.4. Regulatory T Cells

Regulatory T cells (Tregs) negatively regulate the activation, proliferation and effectors functions of immune cells to maintain tolerance to self-antigens and to maintain immune homeostasis [160]. It has been shown Tregs (Foxp3^+^, CD3^+^, CD4^+^, CD25^high^, CD127^low^) are significantly decreased in severe SARS-CoV-2 patients, suggesting a possible role in hyper-inflammatory responses in COVID-19 pathogenesis [161,162,163]. Concordantly, the degree of Treg recruitment into the lungs of patients may determine the severity of disease, with patients who succeed in recruiting Treg cells experiencing milder disease.

Foxp3 is the master regulator of Treg cell development and can be induced in activated T-cells to suppress inflammation and excessive immune responses [164]. In severe SARS-CoV-2 infection, Foxp3-mediated negative feedback mechanisms in the lung is impaired, while activated CD4^+^ T cells produce the protease Furin, which facilitates the SARS-CoV-2 viral entry into pulmonary epithelial cells [165]. It was suggested that CD25-expressing activated T cells preferentially differentiate into effector Th1 or Th2 cells, rather than maturing into Foxp3 Tregs, during severe SARS-CoV-2 infection [165,166]. Furthermore, inflammatory cytokines including IL-6 and TNF-α could trigger Foxp3 degradation, suggesting a pathway for modulating Foxp3^+^ Treg-cell stability during inflammation [167]. Other studies demonstrated that TNF-α could inhibit both CD4^+^ CD25^+^ and TGFβ1-induced Tregs, and through protein phosphatase 1, reduce phosphorylation of Ser418 on Foxp3, thus impairing Treg function [168,169]. Based on these observations it is tempting to speculate that the high IL-6 and TNF-α present during the SARS-CoV-2 induced cytokine storm may reduce the suppressive function of Tregs and subsequently lead to increased expression of IL-17 and IFN-γ within the inflamed tissue.

Treg transfers may represent a putative therapy to reduce immunopathology during COVID-19. Adoptive cord blood (CB) Treg therapy has been demonstrated in mice to resolve acute respiratory distress syndrome (ARDS) and SLE [170,171]. CB Tregs maintain suppressive function in an inflammatory milieu and have low risk of converting to RORγt-expressing Th17 cells, a plasticity concern that plague peripheral blood Tregs [172]. Similar to these findings, in a small trial CB Tregs were given to two COVID-19 patients with ARDS, resulting in a significant reduction of inflammatory markers including IL-6, IL-12, TNF-α, IFN-γ and MCP-1, without adverse reactions [173]. Thus, understanding Treg function in COVID-19, and identifying mechanisms to regulate the balance between Tregs and other T helper subtypes, may provide new therapeutic options for ARDS associated with COVID-19 infection.

### 4.5. Memory T Cells in COVID-19

Tissue resident memory T cells activate innate cells, recruit additional memory T cells, and enhance immune protection against pathogens through the secretion of cytokines such as IFN-γ and CXCL9-11 [174,175]. SARS-CoV-specific memory T cells persist up to 6 years post-infection, and importantly, these T cells are reported to cross-react with SARS-CoV-2 17 years later, with IFN-γ responses to SARS-CoV N protein still present [176]. Additionally, SARS-CoV-2 specific stem cell-like memory T cells (CCR7^+^ CD127^+^ CD45RA^−/+^ TCF1^+^) have been observed in convalescent and asymptomatic individuals [177]. These have been suggested to provide some protection against SARS-CoVs re-infection [102,178]. While the long-term effect of SARS-CoV-2 on immunological memory remains unclear, SARS-CoV infection may provide some insights. SARS-CoV specific CD4^+^ T cells in moderate to severe cases had a central memory phenotype (CD27^+^/CD45RO^+^), with high production of IL-2, TNF-α, and IFN-γ. In a similar fashion, CD8^+^ T cells in these patients were predominantly of a memory phenotype, exhibiting a high frequency of TNF-α, IFN-γ, and CD107a. Moreover, tissue resident memory CD8^+^ T cells could block the spread of viral disease from upper to lower respiratory tract in influenza A infection, preventing the development of severe pulmonary disease [179]. While these data point towards the possibility of long-term T cell memory against SARS-CoV-2, a recent study identified lower levels of memory T cells in SARS-CoV-2 patients than in SARS-CoV patients [135,142]. Thus, further studies are needed to better characterize the formation and persistence of memory T cells in response to SARS-CoV-2 infection.

### 4.6. mTOR Immunoregulation: A Novel Intervention Strategy

The mammalian target of rapamycin (mTOR) is a protein kinase activated by phosphatidylinositol 3-kinase signaling. mTOR is the major component of at least two following multiprotein complexes, mTOR complex 1 (mTORC1) and mTORC2, the former which acts as a nutrient sensor and the latter which regulates proliferation and migration [180]. The mTOR pathway plays crucial roles in B cell development. mTORC1 controls the expression of Bcl6, regulating the fate of B cells within the germinal center, with blockade of mTORC1—mTORC1-deficient B cells—decreasing antigen-specific memory B cell and plasma cell populations following B cells activation after influenza immunization [181]. Consistent with other studies, it has been shown that the ATP-competitive mTOR inhibitors sirolimus/rapamycin suppress early development of B-cells in germinal centers, resulting in reduced Tfh formation during acute viral infections [182,183]. Rapamycin treatment prevents murine B-cell proliferation induced by anti-IgM and suppresses B cell growth but not survival [184]. Interestingly, analysis of SARS-CoV-2 protein interactions has determined that the N protein of SARS-CoV-2 binds to targets of mTORC1 including La-related protein 1 (LARP1) [132,185]. LARP1 is an important mTORC1 regulator that functions as a scaffolding protein, facilitating mTORC1-dependent phosphorylation of its substrate proteins [185]. Rapamycin was found previously to disrupt LARP1 and mTORC1 binding, reducing replication of MERS-CoV by ~60% in vitro [186]. Thus, we hypothesize that ADE could be prevented in patients if memory B cell activation is blocked during the early stages of disease in high-risk COVID-19, thus delaying the development of SARS-CoV-2 reactive antibodies. Additionally, rapamycin improves the quality and magnitude of viral-specific CD8^+^ T cell responses in rhesus macaques following vaccination [187]. So early application of mTOR inhibitors in high-risk SARS-CoV-2 patients may be a promising approach to minimize ADE, enhance T cell responsiveness, and thus reduce mortality. Additionally, Sirolimus blocks the expression of viral proteins and virion release in ICU patients infected with the H1N1 influenza virus [188]. Thus, targeting mTOR may both beneficially modulate the immune response while simultaneously inhibiting viral reproduction [189].

## 5. Conclusions

COVID-19 is the defining pandemic and public health emergency of the past two decades. The SARS-CoV-2 virus is readily transmissible and has a host of mechanisms it uses to compromise the immune system. Immunopathology accounts for much of the pathology experienced by COVID-19 patients, and therapies that prevent immune dysregulation are leading candidates to limit damage from COVID-19. At this time there are no approved drugs or vaccines specifically targeting the SARS-CoV-2 virus, making immunomodulating agents such as glucocorticoids and steroids the leading treatment choice [190]. Developing a functional vaccine that provides durable protection will require an understanding of the adaptive immune response to SARS-CoV-2, and the immunoevasion mechanisms employed by SARS-CoV-2 to avoid adaptive immunity. Given that NK and T cells show an exhausted phenotype in COVID-19 patients, studies unraveling the function of virus-specific effector T/B cells and NK cells could give us insight in understanding the involved impaired immune mechanisms in the disease progression at early disease stages. Moreover, dysregulated or over-exuberant inflammatory responses in COVID-19 contribute to a heightened pro-inflammatory milieu in patients [15], and future investigation in anti-inflammatory elements such as Tregs might be beneficial in understanding the virus pathogenesis. Studies from patients with mild-moderate symptoms and identifying immune markers, in theory, could be used to predict which patients are at the risk of developing severe cases of disease, allowing for earlier intervention. The identification of T and B cell epitopes and evaluating the titration and duration of specific antibody-mediated and cell-based immunity will assist in the development of new antibody therapies and vaccine candidates. While there remains large gaps in our knowledge of COVID-19, a better understanding of the adaptive immune response against this virus, and the mechanisms by which SARS-CoV-2 interacts with adaptive immune cells, will help us combat this disease more efficiently.
